# *Tspyl2* Loss-of-Function Causes Neurodevelopmental Brain and Behavior Abnormalities in Mice

**DOI:** 10.1007/s10519-015-9777-8

**Published:** 2016-01-29

**Authors:** Qi Li, Siu Yuen Chan, Kwun K. Wong, Ran Wei, Yu On Leung, Abby Y. Ding, Tomy C. K. Hui, Charlton Cheung, Siew E. Chua, Pak C. Sham, Ed X. Wu, Grainne M. McAlonan

**Affiliations:** Department of Psychiatry, The University of Hong Kong, Hong Kong SAR, China; State Key Laboratory for Cognitive and Brain Sciences, The University of Hong Kong, Hong Kong SAR, China; HKU-SIRI, The University of Hong Kong, Hong Kong SAR, China; Department of Paediatrics and Adolescent Medicine, The University of Hong Kong, Hong Kong SAR, China; Institute of Basic Medicine, Shandong Academy of Medical Sciences, Jinan, China; Medical Physics and Research Department, Hong Kong Sanatorium and Hospital, The University of Hong Kong, Hong Kong SAR, China; Genome Research Centre, The University of Hong Kong, Hong Kong SAR, China; Laboratory of Biomedical Imaging and Signal Processing, The University of Hong Kong, Hong Kong SAR, China; Department of Electrical and Electronic Engineering, The University of Hong Kong, Hong Kong SAR, China; Department of Forensic and Neurodevelopmental Science, Institute of Psychiatry, Psychology and Neuroscience, King’s College, London, UK

**Keywords:** *Tspyl2*, Prepulse inhibition, Locomotion, Lateral ventricles, MRI

## Abstract

Testis specific protein, Y-encoded-like 2 (TSPYL2) regulates the expression of genes encoding glutamate receptors. Glutamate pathology is implicated in neurodevelopmental conditions such as autism spectrum disorder, attention deficit hyperactivity disorder (ADHD) and schizophrenia. In line with this, a microduplication incorporating the *TSPYL2* locus has been reported in people with ADHD. However, the role of *Tspyl2* remains unclear. Therefore here we used a *Tspyl2* loss-of-function mouse model to directly examine how this gene impacts upon behavior and brain anatomy. We hypothesized that *Tspyl2* knockout (KO) would precipitate a phenotype relevant to neurodevelopmental conditions. In line with this prediction, we found that *Tspyl2* KO mice were marginally more active, had significantly impaired prepulse inhibition, and were significantly more ‘sensitive’ to the dopamine agonist amphetamine. In addition, the lateral ventricles were significantly smaller in KO mice. These findings suggest that disrupting *Tspyl2* gene expression leads to behavioral and brain morphological alterations that mirror a number of neurodevelopmental psychiatric traits.

**Electronic supplementary material:**

The online version of this article (doi:10.1007/s10519-015-9777-8) contains supplementary material, which is available to authorized users.

## Introduction

*Testis Specific Protein, Y*-*encoded Like 2* (*TSPYL2*) is X-linked and expressed in both the developing and adult brain (Lin et al. 2006). It has been independently identified by a number of groups, and variously named: NP79 (Sun et al. 2002), CINAP (Wang et al. 2004), CDA1 (Chai et al. 2001), and DENTT (Ozbun et al. 2001). The gene is known to play a role in cell proliferation (Li and Lau 2008), transforming growth factor beta 1 signaling pathway (Ozbun et al. 2001), and synaptic function (Wang et al. 2004; Kristiansen et al. 2010). For example, by binding Calcium/Calmodulin-dependent serine protein kinase (CASK), TSPYL2 forms a complex with the T-box transcription factor T-brain-1 (Tbr1) (Wang et al. 2004). Tbr1 drives the expression of numerous genes critical for neurodevelopment including *N*-methyl-d-aspartic acid (NMDA) receptor subunit 2b (NR2B) and reelin (Wang et al. 2004). We have previously shown that *Tspyl2* loss-of-function mice generated on a 129 Sv/Ev background have reduced expression of NR2B (Tsang et al. 2014), though others have reported that this is not evident on a C57BL/6 background (Chung et al. 2011). Consistent with a possible role for *Tspyl2* pathways in neurodevelopment, Xp11.2 microduplication incorporating the *TSPYL2* locus has been reported in male patients with ADHD (Moey et al. 2015); human *TSPYL2* is close to *ALAS2*, a marker linked to schizophrenia in a linkage study of 34 families (Dann et al. 1997); and mutations in *CASK* are linked to learning disability (Hackett et al. 2010; Tarpey et al. 2009).

Individuals with neurodevelopmental disorders have a range of behavioral abnormalities including impaired prepulse inhibition of startle (PPI) (Ornitz et al. 1992; Castellanos et al. 1996; Ornitz et al. 1999; Braff et al. 2001; McAlonan et al. 2002; Roussos et al. 2015) and differences in activity and sensitivity to amphetamine (AMPH) (Hart et al. 2014; O’Daly et al. 2011; Strakowski et al. 1997). They also have difficulties with social interaction and may have repetitive or ‘obsessional’ behaviours (Alessandri 1992; Lord et al. 2000; Pinkham et al. 2003; Russell et al. 2005). As glutamate pathways are thought to contribute, at least partly, to this range of behaviors (Hokyo et al. 2010; Li and Wolf 1999; Zhang et al. 2007), we directly tested the prediction that *Tspyl2* loss-of-function on a 129 Sv/Ev background would disrupt these behaviors. We also examined the impact of *Tspyl2* loss-of-function on regional brain volumes.

## Materials and methods

### Mice

Animals from the breeding colony were acclimatized to reversed light–dark cycle (light off: 7A.M. to 7P.M.) for 4 weeks before testing. All experiments described in the present study occurred during the dark phase.

Male mice with a loss-of-function mutation of the *Tspyl2* gene (*Tspyl2*^*m/Y*^), designated knockout (KO) and their wildtype (WT) littermates were used throughout the study. The generation of KO mice on a pure 129 Sv/Ev genetic background has been fully described previously, with the loss of *Tspyl2* transcripts (Tao et al. 2011) and protein (Tsang et al. 2014). When animals from the breeding colony became available they were entered into in vivo testing. Eight batches of mice were generated over time and at least 2 different batches per group (WT/KO) were used for most of the behavioural tests. However, only one batch of mice was available for the marble burying test and three-chamber social approach test. Behavior tests were performed as follows: PPI, WT and Tspyl2 KO: N = 14 of each genotype from four different batches of mice. Four other batches were used for the remaining tests and sub-grouped as shown in Table 1. Magnetic resonance imaging (MRI) was carried out in a total of 13 KO mice and 12 for WT mice, 5 of these were completely naïve i.e. they had no behavioral testing before the scan; 8/7 of these mice were from batch I (please see Table 1).

**Table 1:** Batch, number and age of male mice in wildtype (WT) and *Tspyl2* ^*m/Y*^ knockout (KO) mice for each test

Batch		Experiments	No. of wild type (WT)	No. of Tspyl2^m/Y^ (KO mice)	Age (postnatal days)
1–4		Prepulse inhibition	14	14	150
I		Marble burying	8	8	158
I + II		Reciprocal social interaction	15	15	158–172
I + III		MRI	12	13	180
II		Social approach (three-chamber apparatus)	7	7	166
II + IV		Open field test and amphetamine-induced locomotor activity	13	15	188

### Behavior tests

#### Marble burying

Mice were introduced to a clean, sterilized large plastic rat housing cage (42.5 cm L × 26.6 cm W × 18.5 cm H) filled with bedding to a depth of 5 cm and topped with 20 marbles evenly spaced apart in four rows of 5 marbles for 30 min (Gould et al. 2011). A marble was considered to be ‘buried’ when it was two thirds covered by bedding. The number of marbles buried by each mouse was calculated at the end of each test. To control for odor cues, the bedding was changed and marbles were thoroughly cleaned with liquid soap and ethanol between each test.

### Sociability

#### Social approach (three-chamber apparatus)

The social testing apparatus was a rectangular three-chamber box made by the Physiology Work Shop, University of Hong Kong. Each chamber was 20 cm W × 40.5 cm L × 22 cm H and dividing walls were made from clear Plexiglas with small openings (3.5 cm in diameter) allowing access into each chamber. The chambers of the social apparatus were cleaned and fresh bedding was added between consecutive subjects (Moy et al. 2004). The test mouse was first placed in the middle chamber and allowed to explore the three chambers for 10 min. After habituation, an unfamiliar WT mouse (stranger 1) was introduced in a side chamber. The stranger mouse was enclosed in a cylinder (10.5 cm D × 15 cm H) with small holes evenly spaced over the entire surface of the cylinder. An identical empty cylinder was placed in the opposite chamber. Finally, while stranger 1 remained in its cylinder, a new unfamiliar WT male (stranger 2) was placed in the cylinder in the opposite side chamber. All the ‘strangers’ used were unfamiliar, of similar age and were the same sex as the target mouse. Data collection was performed using the EthoVision XT 7.1 tracking system (Noldus Technology, The Netherlands) and the time spent in each chamber was recorded.

#### Reciprocal social interaction (one-chamber apparatus)

The mice tested in the 1-chamber apparatus were housed together prior to testing the social interaction of each individual animal with an unfamiliar ‘stranger’ WT animal of the same sex. Social interaction testing was performed in a 30 cm L × 30 cm W × 30 cm H white plastic arena. Activities were video-recorded by an overhead camera for 10 min. Time spent during the interaction (nose to nose sniffing, nose to anogenital sniffing, and following) with the stranger was recorded by a researcher who was blind to group assignment (Silverman et al. 2010).

#### Prepulse inhibition (PPI) of the acoustic startle response

The procedures and testing parameters for evaluation of PPI have been fully described previously (Li et al. 2009). In brief, the PPI paradigm was conducted using startle chambers for mice (San Diego Instruments, San Diego, CA, USA). In a test session, a mixture of pulse-alone (100, 110, 120 dB), prepulse-plus-pulse (3 prepulse options × 3 pulse options), prepulse-alone (71, 77, 83 dB), and no-stimulus (background noise, 65 dB) trials were presented. Animals were acclimatized for 2 min prior to the first trail. The first 6 trials were 2 pulse-alone trials of three different pulse intensities. Then followed by 10 blocks of 16 trials in pseudorandom order, each block comprised 3 pulse-alone trials, 3 prepulse-alone trials, 9 prepulse-plus-pulse trials, and 1 no-stimulus trial, with the variable intertrial interval (ITI) of a mean of 15 s (10-20 s). The whole session ended with a final block of 6 pulse-alone trials as in the first block. PPI was calculated by the following formula: 100 % × [1 − (mean reactivity on prepulse-plus-pulse trials/mean reactivity on pulse-alone trials)].

#### Spontaneous locomotor activity in the open field

The apparatus comprised 4 identical cubes made of Plexiglas with a white opaque bottom, each measuring 40 cm W × 40 cm L × 40 cm H. In the middle of the floor a central arena (13.5 cm × 13.5 cm) was demarcated by a red line (Meyer et al. 2005). Each mouse was gently placed in the centre of the appropriate arena and allowed to explore undisturbed for 30 min to adapt to the environment. The images were captured by an overhead camera and analyzed using Ethovision tracking system (VersionXT 7.1).

#### Locomotor response to saline (control) and AMPH

Immediately after the open field test, each animal was gently removed from the arena and 5 ml/kg 0.9 % NaCl was administered intraperitoneally. The mice were then returned to the arena for a further 30 min. Afterwards, they were carefully removed and given an intraperitoneal injection of AMPH and returned for a final 60 min. AMPH sulfate, (Sigma–Aldrich) was dissolved in 0.9 % NaCl solution on the day of testing to obtain 2.5 mg/kg, in a volume of 5 ml/kg. This low dose was selected based on evidence that it typically elicits a measureable locomotor response in mice, with minimal stereotypic or ataxic behaviors (Meyer et al. 2005; McNamara et al. 2006). Locomotor activity was evaluated after saline and AMPH injection and indexed by the distance traveled during successive 10-min bins.

#### MRI

In vivo MRI scanning took place at 180 day old in a 7 T scanner with a maximum gradient of 360 mT/m (70/16 PharmaScan, Bruker Biospin GmbH, Germany). Animals were anesthetized during scanning with isoflurane/air mixture at 3 % for induction and 1.5 % for maintenance via a nose cone. A quadrature RF coil with 23 mm inner diameter was used. A set of scout images [T2-weighted: Effective TE = 38.71 ms, TR = 4668 ms, No of Average = 6, Rare Factor = 8, Acquisition Matrix = 256 × 256, FOV = 25 × 25 mm, Slice thickness = 0.25 mm, Scan Time = 11 m12 s in axial orientation were acquired in each animal. This sequence took less than one hour (Li et al. 2009).

#### Region of interest (ROI) measurement

Manual measurements were done using InsightITK-Snap (http://www.itksnap.org/) by a single rater, who was blind to subject group membership. Total brain volume was measured from a mask that delineates brain tissue from the skull by using semi-automatic, region growing “3D-snake” method. Lateral ventricles, cerebral cortex, hippocampus and cerebellum were delineated according to previously described boundaries and landmarks in our own and others’ studies (Li et al. 2009). Voxels within the ROIs were highlighted slice-by-slice with the “Paint bush” tool. Left and right sides of ROIs were labeled separately.

#### Statistical analysis

Marble burying behavior, and MRI were analyzed using independent samples *t* test. Data from the three components of the social behavior test (habituation, sociability and social novelty) were analyzed by using a chamber (stranger 1 side or the opposite side) × 2 genotypes (WT, KO) univariate general linear model (GLM). Where data was not normally distributed, the values were transformed or non-parametric testing was adopted. In reciprocal social interaction, Mann–Whitney non-parametric testing was applied. In PPI, percentage PPI (% PPI) was analyzed using a 2 × 3 × 3 (genotype × prepulse level × pulse level) repeated measures GLM, and reactivity to pulse-alone trials and prepulse-alone trials was analyzed using 2 × 3 (genotype × pulse level) and 2 × 3 (genotype × prepulse level) GLM, respectively. In the open field test/locomotor responses to saline and AMPH, total distances were analyzed using a (genotype × 10-min bins) GLM. In addition, the number of entries in the central arena during the open field test was analyzed using Mann-Whitney non-parametric tests in the open filed test.

## Results

### Behavioral tests

#### Marble burying

Marble burying can be considered a measure of repetitive digging behavior (Thomas et al. 2009). In the marble burying test, *Tspyl2* KO mice tended to bury more marbles than WT controls, although this difference did not reach statistical significance (t = −1.85; *p* = 0.085). (Please see Supplementary Fig. 1).

#### Social approach (three-chamber apparatus)

There was no main effect of genotype on the time spent exploring the whole chamber; and there was no main effect of chamber side during the habituation period. When stranger 1 was introduced, there was however a main effect of chamber [*F*(1, 22) = 71.249, *p* < 0.01]. *T*-tests confirmed that both WT and KO mice had a significant preference for the chamber containing stranger 1 rather than the empty chamber (WT, t = 6.826, *p* < 0.001; *Tspyl2* KO, t = 5.248, *p* < 0.001). When stranger 2 was introduced, there was no main effect of chamber, or genotype, nor any interaction between genotype × chamber sides. Both *Tspyl2* KO and WT mice spent a similar time in the chamber containing stranger 1 and the chamber with stranger 2. (Please see Supplementary Fig. 2a–c).

#### Reciprocal social interaction (one-chamber apparatus)

Both WT and *Tspyl2* KO behaved in a social manner and there was no significant group difference in time spent on nose-to-nose, nose-to-anogenital region sniffing and following. (Please see Supplementary Fig. 2d).

#### PPI

**Fig. 1:**
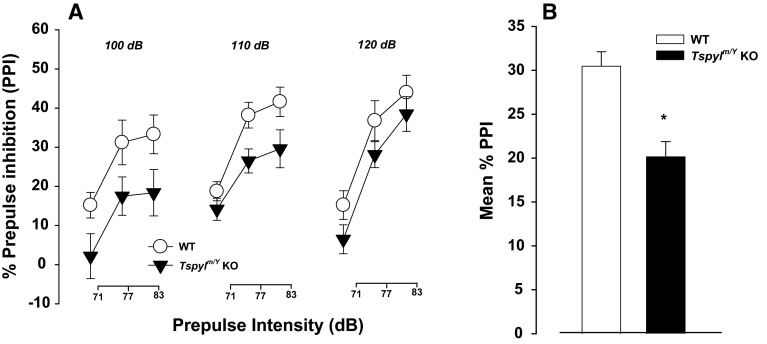
PPI in WT and *Tspyl2* ^*m/Y*^ loss-of-function mice. **a** % PPI at three different pulse levels (100, 110, and 120 dB) and three different prepulse levels (71, 77, and 83 dB). **b**. The *bar plot* depicts mean% PPI across all prepulse and pulse stimuli used in two groups. All values are mean ± SEM. *p < 0.05

Sensorimotor gating was assessed using the PPI of acoustic startle reflex. *Tspyl2* KO had significantly lower % PPI compared to WT mice (Fig. 1a), main effect of genotype [*F*(1, 26) = 5.6, *p* < 0.05] (Fig. 1a, b).

There were no significant group differences in reaction to prepulse-alone trials and pulse-alone trials (Supplementary Fig. 3). Therefore, the PPI attenuating effects of *Tspyl2* KO mice were not due to changes in general startle reactivity and/or prepulse detection, reflecting a genuine disruption of sensorimotor gating function.

#### Spontaneous locomotor activity in the open field

The distance moved in 10 min bins during the habituation period in the open field was taken as a measure of spontaneous locomotor activity. The *Tspyl2* KO mice were marginally more active than WT controls, though this difference did not quite reach significance [*F*(1, 78) = 4.13, *p* = 0.054]. Total distance travelled in 30 min in the open field was shown in Supplementary Fig. 4. There was no group difference in the number of entries in the central arena (Please see Supplementary Fig. 4).

#### ‘Non-specific’ locomotor response to saline injection

**Fig. 2:**
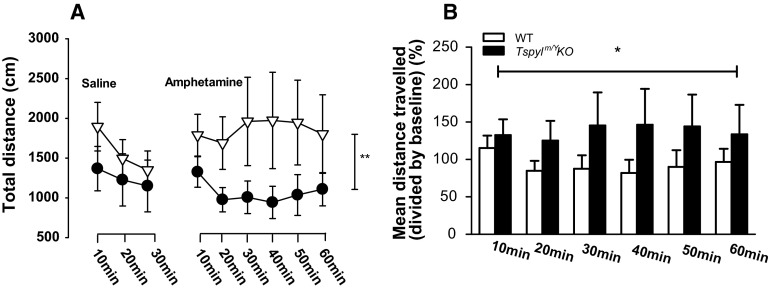
Locomotor response to amphetamine challenge in the open field test. **a** Saline administration (30 min, *left panel*) and amphetamine (Amph, 2.5 mg/kg) (60 min, *right panel*) challenge by timebins (10 min). **b** Distance travelled after Amph challenge (60 min) as a percentage of distance travelled in the final 10-min after saline injection. *Tspyl2* KO mice had significantly greater sensitivity to Amph when compared to WT controls. All values are mean ± SEM. *p < 0.01; **p < 0.001

There was no significant group difference in distance moved in 10 min bins following saline injection (Fig. 2a). Thus the trend towards baseline group differences in locomotion was no longer apparent.

#### Locomotor response to AMPH

Following AMPH injection, the total distance moved within the entire arena was analyzed using 2 × 6 (genotype × 10-min bins) ANOVA. There was a significant main effect of genotype [*F*(1, 156) = 12.464, *p* < 0.001]. The total distance moved was significantly greater in the *Tspyl2* KO group relative to WT mice (Fig. 2a). To confirm the relative (to saline) increase in locomotion after AMPH challenge in each KO mice, the distance travelled in each 10 min bin following AMPH injection was divided by the distance travelled in the final 10 min after saline injection. This analysis confirmed that *Tspyl2* KO mice had significantly greater sensitivity to AMPH when compared to WT controls [F (1,156) = 7.034, *p* < 0.01] (Fig. 2b); estimated marginal means, WT = 92.715 ± 12.477 (SEM) and KO = 137.926 ± 11.615 (SEM).

#### MRI

**Fig. 3:**
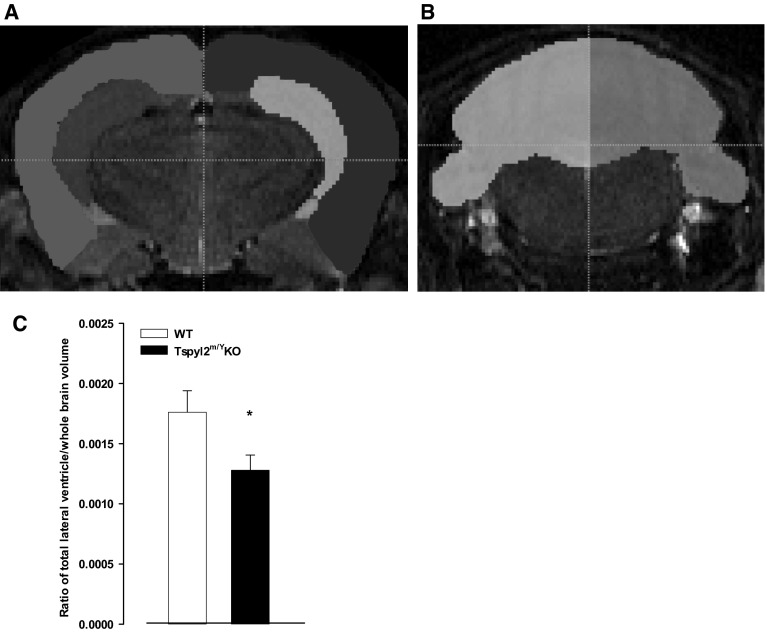
ROI segmentations of MRI and measurement of lateral ventricles. **a** *Dark blue* and *yellow, left* and *right* hippocampus. *Red* and *green*, *left* and *right* lateral ventricle. *Pink* and *light blue*, *left* and *right* neocortex. **b** *Brown* and *purple*, *left* and right side of cerebellum. **c** Ratio of total lateral ventricle/whole brain volume. *p < 0.05. All values are mean ± SEM

There were no differences in regional brain volumes between the naïve mice and mice that had behavior testing in each group (data not shown). Therefore all animals with MRI data were considered together in the analyses of group differences. ROI tracing of the lateral ventricles, cerebral cortex, hippocampi and cerebellum are shown in Fig. 3a, b. Regional volumes were corrected by dividing the raw measure by the total brain volume. The total volume of the lateral ventricles (corrected) was significantly lower in *Tspyl2* KO mice (t = 2.245, *p* < 0.05, Fig. 3c) as was the volume of the left and right lateral ventricle. However, there was no significant difference in the volume of the cerebral cortex, hippocampi or cerebellum (Please see Supplementary Table 1).

## Discussion

In this study, we found that loss-of-function of the *Tspyl2* gene on a 129 Sv/Ev background led to sensorimotor gating impairment, mild hyperactivity and hyper-sensitivity to the dopamine agonist AMPH. However, *Tspyl2* loss-of-function did not alter social interaction as tested here, nor did it cause gross abnormalities in volume of the whole brain, hippocampus or cerebellum, although smaller ventricular volumes were observed in KO mice.

### Behavioral effects of *Tspyl2* loss-of-function

#### PPI

Disruption of PPI is widely accepted as an endophenotype of schizophrenia and autism; it is also reported in individuals with ADHD complicated by comorbid enueresis (Ornitz et al. 1999). Importantly, it is one of the few paradigms in which humans and rodents are tested in similar ways (Geyer et al. 2002). PPI reflects a sensorimotor gating function which is a precognitive process to prevent sensory overload and cognitive fragmentation (Geyer et al. 2002). The findings from the experiments on humans and rodents indicate that dopaminergic manipulations modify prepulse effects on startle response (Talledo et al. 2009; Mosher et al. 2015; Depoortere et al. 1997). However, alongside this long-standing model of dopamine dysfunction in sensorimotor gating, there is growing evidence of an interaction between dopamine and glutamate in sensorimotor gating (Wan et al. 1995), and indeed the importance of glutamate systems in the pathophysiology of schizophrenia (Coyle 2006), autism (Lee et al. 2015) and ADHD (Maltezos et al. 2014). *Tspyl2* loss-of-function mice have reduced expression of NR2B (Tsang et al. 2014); and inhibition of NR2B subunit containing NMDA receptors is known to disrup t PPI (Spooren et al. 2004). Thus, PPI impairment in *Tspyl2* loss-of-function mice may be a consequence of glutamate signaling disruption.

#### Locomotor activity and response to AMPH

A trend towards increased baseline locomotor activity was observed in our *Tspyl2* KO mice. This could also be at least partly explained by glutamate dysfunction. For example, increased locomotor activity has been reported in NR2B KO animals (Badanich et al. 2011). However, NR2B loss may not be the full story as greater activity has also been reported following *Tspyl2* KO on a different background strain that does not alter NR2B (Chung et al. 2011).

It is important to note that, although the KO mice were marginally more active than WT in the first 30 min in the open field, this activity difference had resolved prior to amphetamine challenge. Specifically, there was no difference in activity between WT and KO following a saline control injection. Thus we do not think that baseline activity differences could explain the differential response to amphetamine. Rather, glutamate dysfunction in *Tspyl2* loss-of-function mice may also have contributed to their AMPH sensitivity. For example, genetic disruption of NR2B (typically lowered in these animals) can induce behavioral sensitivity to AMPH (Mao et al. 2009).

Our observation that the WT 129 SV/Ev animals had almost no response to amphetamine challenge in our study is consistent with previous reports (Chen et al. 2007; Good and Radcliffe 2011; Zhang et al. 2015). These studies agree that the 129 mouse strain has one of the lowest responses to amphetamine challenge compared to other strains. As we elected to use a particularly low dose of AMPH challenge (2.5 mg/kg) to avoid the potential confounds of stereotypy, the lack of response to AMPH in WT animals is therefore not surprising.

#### Social function

Our *Tspyl2* KO mice had preserved social functioning, at least in the tests employed in this study. However, assessment of ecologically valid social behavior in the mouse is challenging, as is their translation to the human condition.

### Anatomical effects of *Tspyl2* loss-of-function

*Tspyl2* loss-of-function did not have gross effects on brain anatomy. The main finding was smaller ventricular volumes in the loss-of-function animals relative to WT controls; the volumes of cortex, hippocampus and cerebellum volumes were unaltered. Across neurodevelopmental conditions, findings of enlarged ventricles in schizophrenia are reasonably robust (Jaaro-Peled et al. 2010) and animal models have been shown to replicate this feature (Li et al. 2009; Piontkewitz et al. 2011). Also, posterior lateral ventricular enlargement has been reported in ADHD (Lyoo et al. 1996). However, MRI findings in studies of autism are at variance. Young children with autism have enlarged brains which may result from both greater brain tissue volume and greater lateral ventricle volume (Piven et al. 1995). Yet, by the age of 10 years, more recent three-dimensional measurements have found smaller left frontal and occipital ventricular horns in individuals with autism spectrum disorders (Vidal et al. 2008). Preclinically, BTBR mice, an animal model with utility in autism research (McFarlane et al. 2008), has been reported to have smaller lateral ventricles compared to C57BL/6J mice (Meyza et al. 2012). Thus, we cautiously suggest some overlap in anatomical pattern observed in our study with some reports in the autism literature.

## Limitations

In this study we attempted to examine the effect of *Tspyl2* gene KO on a range of behaviors as well as brain anatomy. This was logistically difficult and meant that not every animal in the study was tested in every paradigm. This may have been a particular issue for the marble burying task as the small sample size possibly leads to poor statistical power and the risk of a false negative result. In addition, although the genotype of *Tspyl2* KO mice was confirmed by western blot at the outset of our programme of study (Tsang et al. 2014), we did not repeat the western blot for every litter used in the present study.

## Conclusion

In summary, this study demonstrates that *Tspyl2* KO mice have behavioral and brain anatomical differences that model some features found in neurodevelopmental psychiatric disorders. We have previously shown that this gene regulates NMDA receptor 2A and 2B expression (Tsang et al. 2014). Therefore we suggest that disruption of glutamate signaling may at least partly explain the phenotype observed here, in line with evidence for a critical role of this system in neurodevelopmental psychiatric disorders.

## Electronic supplementary material

Below is the link to the electronic supplementary material.

Supplementary material 1 (DOCX 194 kb)
