# Supplementary material for: Tspyl2 Loss-of-Function Causes Neurodevelopmental Brain and Behavior Abnormalities in Mice
Source: Behav Genet. 2016 Jan 29;46:529–37. doi: 10.1007/s10519-015-9777-8 (PMC4886156; doi:10.1007/s10519-015-9777-8)
Supplement: Supplementary file 1 — Supplementary material 1 (DOCX 194 kb) [file 10519_2015_9777_MOESM1_ESM.docx]

**Supplementary Figure 1**


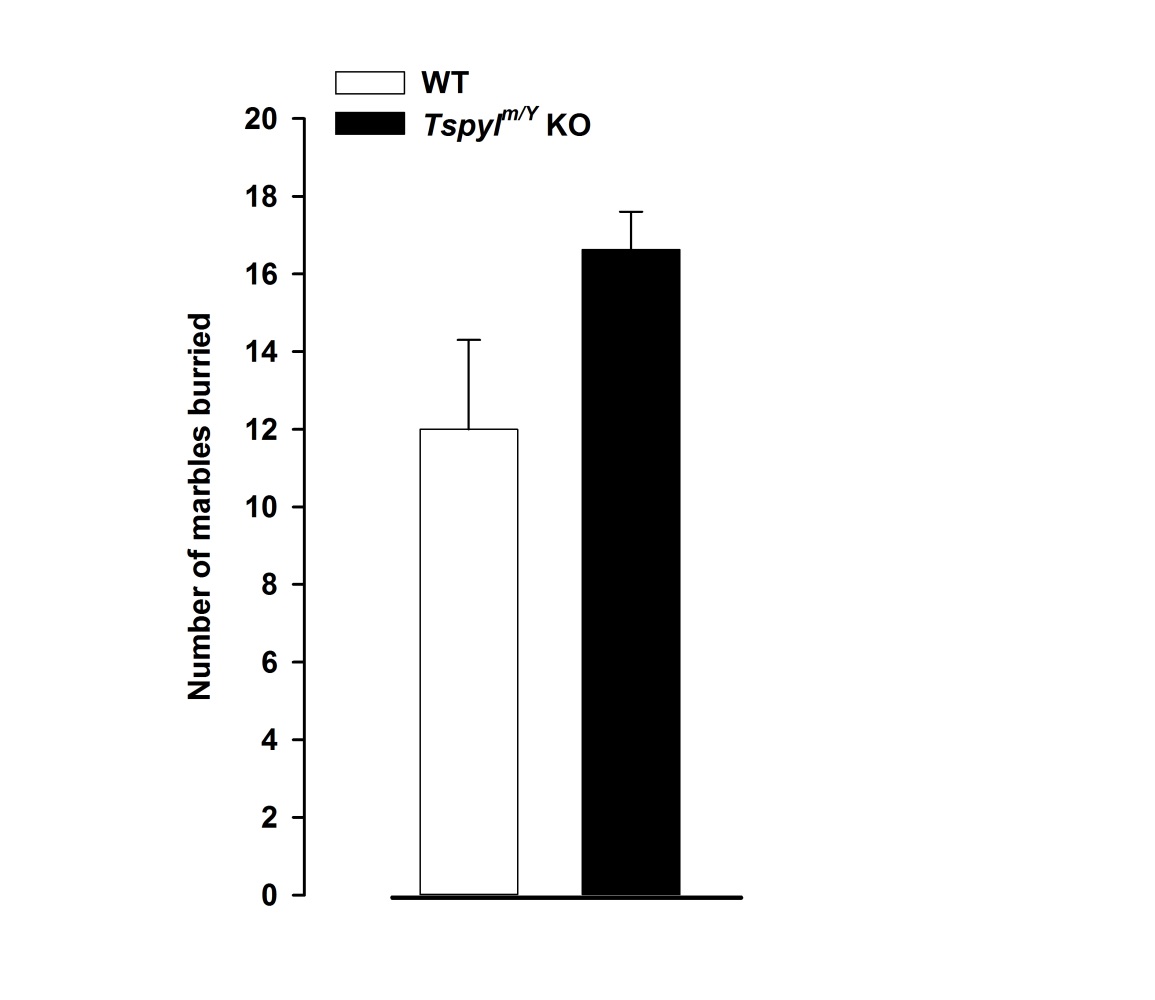


**Fig.1s.** The number of marbles buried in 30 mins. *Tspyl2^m/Y^* KO mice tended to bury more marbles than WT controls, although this difference did not reach statistical significance (*p* = 0.085). All values are mean ± SEM.

**Supplementary Figure 2**

**b.**

**a.**


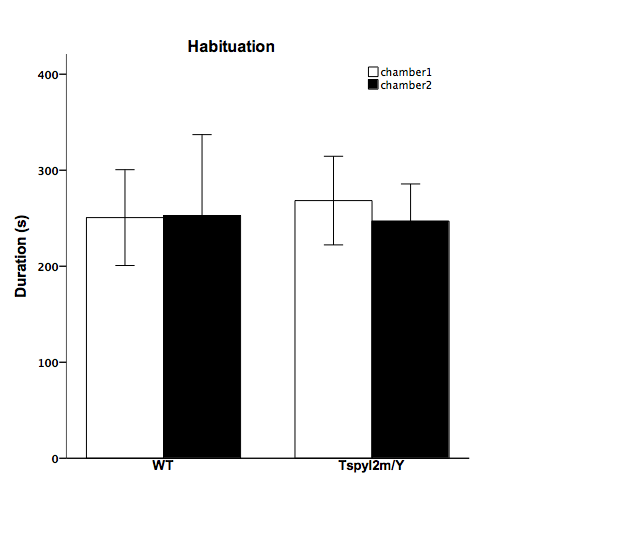

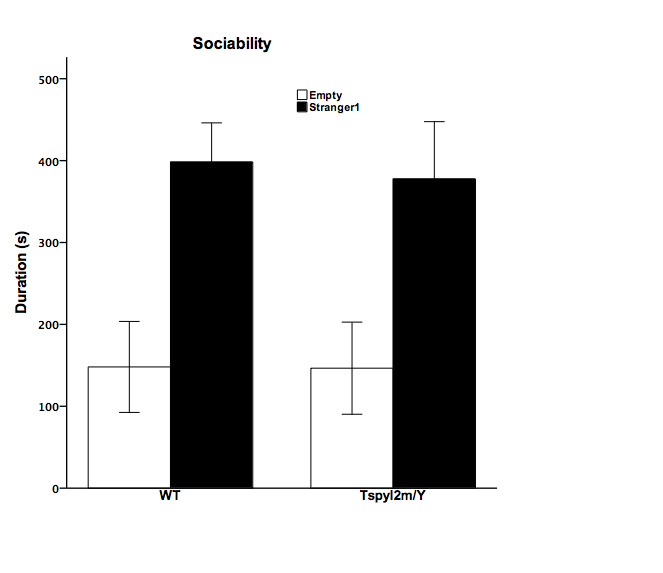


**d.**

**c.**


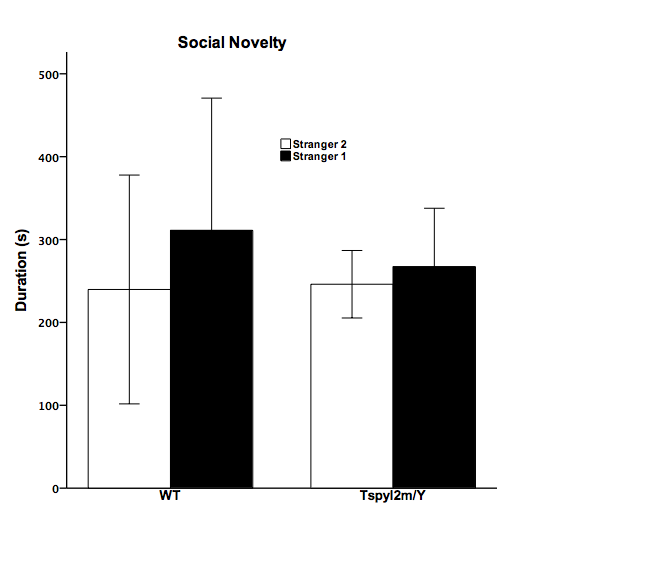

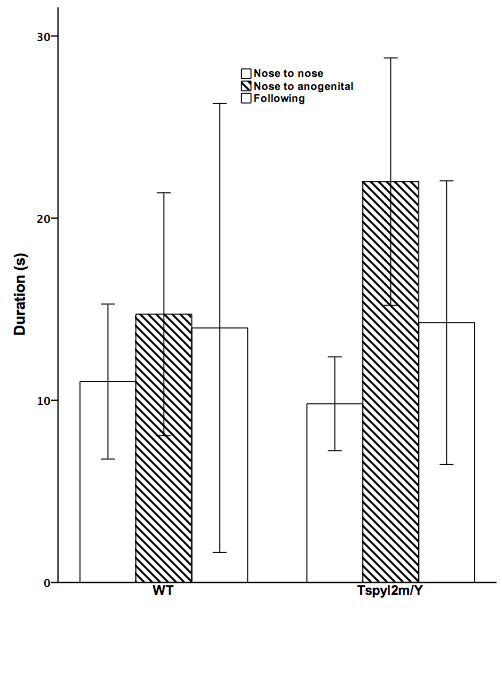


**Fig. 2s. a-c** Duration of time spent in the chambers during the test for ***a.*** habituation; ***b.*** sociability and ***c.*** preference for social novelty for 10 mins in each test by using three-chamber social test apparatus. **d.** Duration of reciprocal social interaction behaviors, including the nose-to-nose sniffs, nose-to-anogenital, and following, was measured manually in wildtype (WT) and *Tspyl2^m/Y^* KO mice. All values are mean ± SEM.

**Supplementary Figure 3**

**b.**

**a.**

**Fig. 3s. a.** Reactivity to prepulse-alone trials including background, 71 dB, 77 dB and 83 dB. **b.** Startle reactivity to pulse-alone trials (100 dB, 110 dB and 120 dB). All values are means ± SEM.

**Supplementary Figure 4**

**b.**

**a.**

**Fig. 4s.** Spontaneous locomotor activity in the open field test. **a.** Spontaneous locomotor activity over 30 mins, the *Tspyl2^m/Y^* KO mice were marginally more active than WT controls, *p* = 0.054. **b.** Entries in central zone over 30 mins. All values are means ± SEM.

**Supplementary Table 1**

**Table1s.** Ratio of brain volume of cortex, hippocampi and cerebellum in WT and *Tspyl2^m/Y^* KO mice.

| Groups | Cortex Vol. | Hippocampi Vol. | Cerebellum Vol. |
| --- | --- | --- | --- |
| WT | 0.071±0.001 | 0.0428±0.001 | 0.1408±0.003 |
| KO | 0.072±0.001 | 0.0422±0.001 | 0.1393±0.002 |

Volume (Vol.) ratio of total cortex or hippocampi or cerebellum vol./whole brain volume. All values are mean ± SEM. WT, wildtype; KO, knockout.
